# Immunoglobulin A Vasculitis After Initiation of Treatment for Tuberculous Pleurisy: A Case Report and Literature Review

**DOI:** 10.7759/cureus.58707

**Published:** 2024-04-22

**Authors:** Yuri Hiramatsu, Kazunori Tobino, Yukari Saito, Shota Sogabe, Yosuke Murakami

**Affiliations:** 1 Respiratory Medicine, Iizuka Hospital, Fukuoka, JPN

**Keywords:** corticosteroid therapy, anti-tuberculosis treatment, tuberculous pleurisy, henoch-schönlein purpura, iga vasculitis

## Abstract

Immunoglobulin A vasculitis (IgAV), also known as Henoch-Schönlein purpura (HSP), is a disease that causes inflammation and bleeding in small blood vessels in the skin, joints, intestines, and kidneys. Although various infections and chemicals are known to be triggers, the underlying cause of IgAV remains unknown. Here, we describe a case of an 86-year-old male patient with IgAV that developed after anti-tuberculosis treatment for tuberculous pleurisy. There have been several case reports implicating *Mycobacterium tuberculosis* and other acid-fast bacterium in the development of IgAV, but only a few case reports implicating anti-tuberculous drugs. This case highlights the importance of recognizing that IgAV can be caused by anti-tuberculous drugs.

## Introduction

Immunoglobulin A vasculitis (IgAV), also known as Henoch-Schönlein purpura (HSP), is a disease that causes inflammation and bleeding in small blood vessels in the skin, joints, intestines, and kidneys. The most prominent feature is a purple rash, usually on the lower legs and buttocks. IgAV can also cause abdominal and joint pain and, rarely, serious kidney damage. IgAV can affect anyone but is most common in children under 10 years of age [1,2]. Although various infections and chemicals are known to be triggers, the underlying cause of IgAV remains unknown. Immunological, genetic, and environmental factors all seem to be involved.

Here, we describe a case of IgAV that developed after anti-tuberculosis treatment. IgAV caused by anti-tuberculous drugs is very rare. Although the pathogenesis is not yet understood, there are a few similar reports, and it is thought that IgAV may be induced by anti-tuberculosis treatment.

## Case presentation

**Figure 1:**
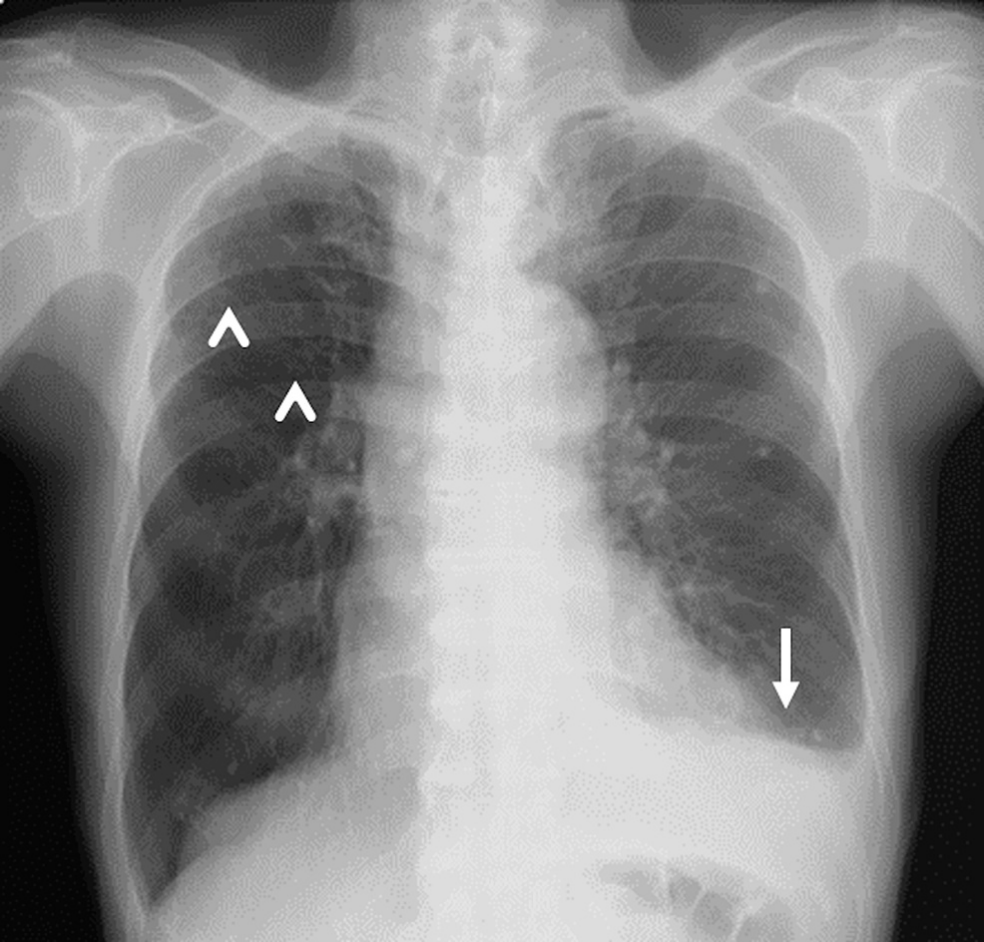
Chest X-ray on admission. Left-sided pleural effusion (white arrow) and multiple nodules in the right upper lung field (white arrowhead) were observed.

An 86-year-old man presented to a previous hospital for shortness of breath and palpitation that persisted for two weeks. A chest X-ray obtained at the hospital showed left-sided pleural effusion with multiple nodules in the right upper lung field (Figure 1), and he was referred to our department for a detailed examination. His medical history included chronic gastritis, constipation, and benign prostatic hyperplasia. He had never smoked and drank a glass of beer every day. His regular medication included tamsulosin, ifenprodil, levocetirizine, Swertia, sodium bicarbonate powder, and picosulfate sodium. His blood pressure was 145/87 mmHg, pulse was 74/minute, and respiratory rate was 18/minute, with an O2 saturation of 98% on room air. Lung auscultation revealed decreased breath sound on his left lower lung field. His cardiovascular examination was normal, and the abdominal examination was unremarkable. There was no edema in his extremities.

**Figure 2:**
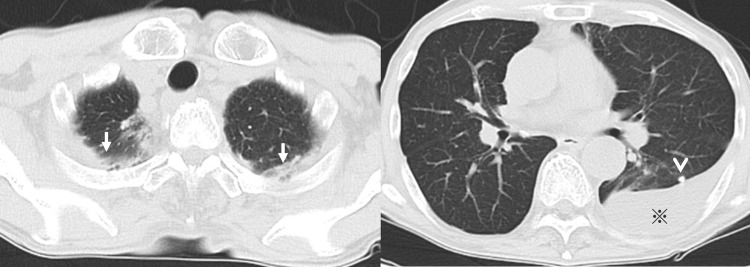
Chest CT image. Old inflammatory changes in the upper lobes of both lungs (white arrows), scattered calcified nodules in both lungs (arrowhead), and an encapsulated pleural effusion (※) in the left pleural cavity were observed.

**Figure 3:**
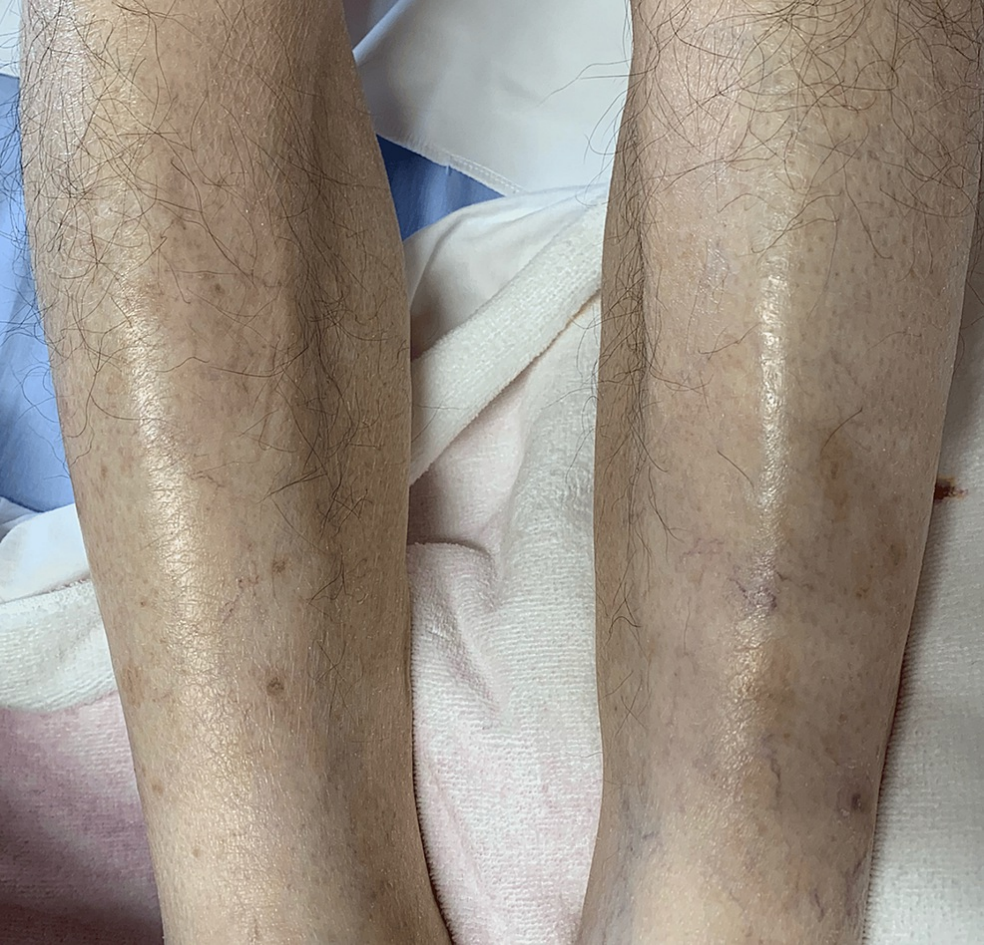
Picture of skin rash on patient's lower extremities at the onset of immunoglobulin A vasculitis (IgAV). Nontender palpable purpura appeared on the patient’s lower extremities.

**Figure 4:**
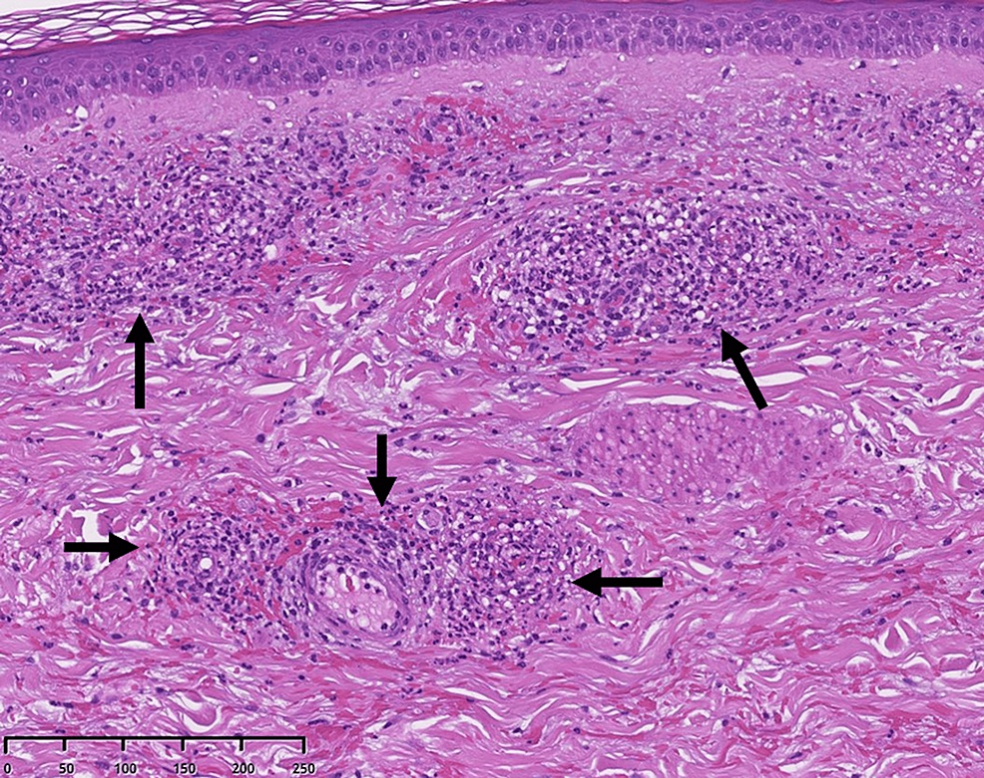
The results of skin biopsy (magnification 200x, hematoxylin and eosin stain). The shallow dermis is accompanied by inflammatory cell infiltration, including neutrophils around the capillaries, and some infiltrate the vessel wall as well (black arrows).

**Figure 5:**
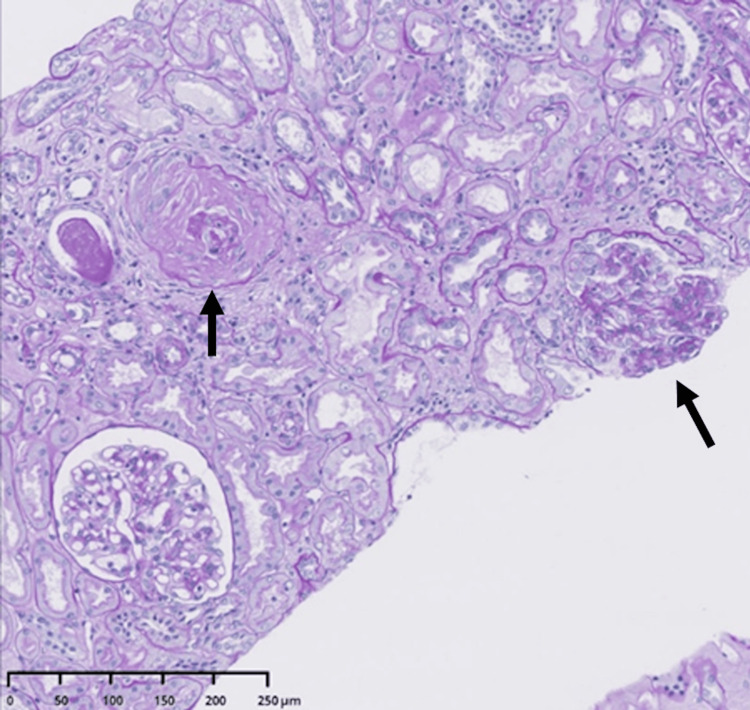
The results of renal biopsy (magnification 100x, periodic acid-Schiff stain). The proliferation of renal glomerular mesangium cells was observed (black arrows).

Computed tomography (CT) of the chest showed old inflammatory changes in the upper lobes of both lungs, scattered calcified nodules in both lungs, and an encapsulated pleural effusion in the left thoracic cavity (Figure 2). He underwent thoracentesis and his pleural fluid was lymphocyte-predominant (75%) exudate with a high adenosine deaminase (ADA) level (121 U/L). No microorganisms, including acid-fast tuberculosis bacilli, were detected in his pleural effusion and sputum smear and culture. Tuberculosis polymerase chain reaction testing of pleural fluid was performed using Cobas 5800 MTB (Roche Diagnostics, Indianapolis, IN) with negative results. However, the tuberculosis blood T-SPOT TUBERCULOSIS interferon γ release assay was positive. Based on the patient's clinical presentation, imaging findings, and pleural fluid analysis, the results were consistent with tuberculous pleurisy. Blood tests and whole-body CT showed no findings suggestive of disease other than tuberculosis, and a clinical diagnosis of tuberculous pleurisy was made. Although drug resistance testing could not be performed because mycobacteria were not isolated, the standard treatment was administered because the drug resistance rate of *Mycobacterium tuberculosis* in untreated patients in Japan is relatively low. He was started on a three-drug anti-tuberculosis regimen (ethambutol, isoniazid, and rifampicin), excluding pyrazinamide due to his elderly status, as some experts recommend avoiding pyrazinamide in patients over 75 years old, according to the American Thoracic Society/CDC/Infectious Diseases Society of America (ATS/CDC/IDSA) Guidelines for Treatment of Drug-Susceptible Tuberculosis (2016) [3]. On the 16th day of treatment, fever and diffuse rash on his trunk occurred, which was thought to be caused by the anti-tuberculous drug, and the administration was discontinued. The fever persisted even after discontinuation of anti-tuberculous drugs, and the skin rashes on his trunk became non-tender 1-2 mm sized palpable purpura and appeared on bilateral lower legs (Figure 3). No abdominal pain or arthralgia was noted, but proteinuria and hematuria were detected by urinalysis. Therefore, IgAV was suspected, and he underwent skin and renal biopsy (Figures 4, 5). Skin biopsy showed inflammatory cell infiltration, including neutrophils around capillaries in the shallow dermis, with some neutrophils also infiltrating the vessel wall, consistent with leukocytoclastic vasculitis. Renal biopsy revealed diffuse mesangial proliferation with a small increase of mesangial matrix, and seven of 10 glomeruli (70%) showed cellular crescent formation. Immunofluorescence microscopic examination showed a predominant IgA deposition in the mesangial area.

Based on these findings, the patient was diagnosed with IgAV, and considering the timing of onset, anti-tuberculous drugs were suspected to be the cause. An upper and lower gastrointestinal (GI) endoscopy was performed to examine the GI lesions, but no abnormal findings were found. Steroid pulse therapy (methylprednisolone 1000 mg/day for three days) was started, followed by prednisolone 30 mg/day. Purpura, proteinuria, and hematuria improved immediately after the initiation of the steroid treatment. After confirmation of improvement of vasculitis symptoms (i.e., palpable purpura, proteinuria, and hematuria), anti-tuberculous drugs were re-started one by one, parallel with the steroid treatment. Thereafter, he completed a total of 12 months of anti-tuberculosis treatment without worsening of tuberculosis or IgAV. The steroids were also tapered off and ended 12 months after the start of treatment. Twelve months after the end of treatment, his condition is stable with no relapse of both disease.

## Discussion

Previously published case reports of IgAV after initiation of anti-tuberculous drugs are very rare. IgAV is a leukocytoclastic vasculitis that usually affects children. According to the European League Against Rheumatism/Paediatric Rheumatology International Trials Organisation/Paediatric Rheumatology European Society (EULAR/PRINTO/PRES) IgAV criteria, patients were classified as IgAV if they had purpura or petechial hemorrhage predominantly in the lower extremities (required) and also met one of the following four criteria: abdominal pain; histopathology (IgA); arthritis or arthralgia; renal involvement [4]. Our patient met this criterion and was diagnosed with IgAV. Our patient’s symptoms occurred after the initiation of anti-tuberculous drugs and improved with discontinuation of the drugs and initiation of steroid treatment; therefore, we suspected that his IgAV was triggered by anti-tuberculous drugs.

Some precipitating factors have been reported as follows: infections (including tuberculosis), drugs, malignancy, environmental chemicals, insect bites, trauma, and complement C2 deficiency [5]. Previously reported drugs that were suspected as the cause of IgAV include vaccines, antibiotics, and tumor necrosis factor-alpha (TNF-α) inhibitors. Among antibiotics, IgAV cases were reported for beta-lactams, fluoroquinolones, and macrolides. The median time from drug initiation and the onset of vasculitis was 10 days for antibiotics [6]. It has been suggested that immune complexes are formed in active tuberculosis and that this may be related to the mechanism of vasculitis [7,8]. In addition to tuberculosis, there are several case reports of IgAV after non-tuberculous mycobacterium (NTM) infection [9]. On the other hand, immune complex-mediated vasculitis, which may be caused by anti-tuberculous drugs, such as rifampicin, has also been reported [10]. It means that not only mycobacterium infections but also anti-tuberculous drugs can cause IgAV. To the best of our knowledge, 11 case reports have been published suggesting an association between tuberculosis and IgAV. Seven of these patients developed IgAV after the start of anti-tuberculosis treatment, and the other four had tuberculosis and IgAV symptoms at the same time. Among those who developed IgAV after starting anti-tuberculosis treatment, four of seven cases improved with the use of systemic corticosteroids [11-14], two cases were cured spontaneously with continued anti-tuberculosis treatment [15,16], and the remaining cases improved with discontinuation of anti-tuberculous drugs [17]. On the other hand, of the four patients diagnosed with IgAV prior to anti-tuberculosis treatment, three were cured with anti-tuberculosis treatment alone [18-20], and the remaining one improved with a combination of anti-tuberculosis treatment and systemic corticosteroid treatment [21].

When IgAV develops after anti-tuberculous drugs are started in a tuberculosis patient, as in our patient, it is difficult to distinguish whether the cause is tuberculosis or anti-tuberculous drugs. In addition, it may be due to the host's immune response to the large numbers of *Mycobacterium tuberculosis* rapidly killed by anti-tuberculosis treatment. If this manifested as swelling of the lesions, the appearance of new lesions, and increased pleural effusions, it would be called a paradoxical reaction. To the best of our knowledge, one case of anti-neutrophil cytoplasmic antibody (ANCA)-related rapidly progressive glomerulonephritis that developed during anti-tuberculosis treatment has been reported [22]. In this case, during treatment for drug-sensitive pulmonary tuberculosis, the patient developed a drug paradoxical reaction that worsened his symptoms and radiological features. Subsequently, the patient showed features of severe acute kidney injury and glomerulonephritis, with perinuclear-ANCA and anti-myeloperoxidase antibodies present in the serum. Renal biopsy showed findings consistent with ANCA-associated vasculitis. The patient was successfully treated with both anti-tuberculosis therapy and immunosuppressive therapy (corticosteroids and mycophenolate mofetil), resulting in clinical improvement and recovery of renal function. To the best of our knowledge, no specific reports of IgAV due to the paradoxical reaction of anti-tuberculosis treatment were found. To the best of our knowledge, we have not found any specific reports of IgAV involvement in paradoxical reactions to anti-tuberculosis treatment, and we did not observe symptoms of paradoxical reactions in our patient, but we believe that it is possible as a pathophysiology.

Our patient developed IgAV after starting anti-tuberculous drug therapy, requiring temporary discontinuation of anti-tuberculous drugs and systemic corticosteroid therapy. It is difficult to conclude that anti-tuberculous drugs were the cause of IgAV in our patient because symptoms did not recur after the resumption of anti-tuberculous drugs. It is quite possible that IgAV was masked by the long-term concomitant use of corticosteroids with anti-tuberculous drugs. However, tuberculosis is a disease that cannot be left untreated, and in such cases, the resumption of anti-tuberculous drugs is necessary. Based on our patient and previous reports, we believe that anti-tuberculosis treatment with systemic corticosteroids should be attempted in such cases while resuming and continuing anti-tuberculous drugs. Although this is a rare case, we believe it is a practice that should be shared with clinicians.

## Conclusions

This case highlights the importance of recognizing that IgAV can occur during anti-tuberculosis treatment. Tuberculosis-induced IgAV often improves with tuberculosis treatment, but anti-tuberculous drug-induced IgAV may require steroids for treatment. On the other hand, treatment for tuberculosis should be continued. Further study is needed on the combination of steroids and anti-tuberculous drugs and the timing of the combination.
